# Planned Vaginal Birth or Elective Repeat Caesarean: Patient Preference Restricted Cohort with Nested Randomised Trial

**DOI:** 10.1371/journal.pmed.1001192

**Published:** 2012-03-13

**Authors:** Caroline A. Crowther, Jodie M. Dodd, Janet E. Hiller, Ross R. Haslam, Jeffrey S. Robinson, on behalf of the Birth After Caesarean Study Group

**Affiliations:** 1 Australian Research Centre for Health of Women and Babies (ARCH), The Discipline of Obstetrics and Gynaecology, The University of Adelaide, Adelaide, South Australia, Australia; 2 Faculty of Health Sciences, Australian Catholic University, Melbourne, Victoria, Australia; 3 Department of Neonatal Medicine, The Women's and Children's Hospital, Adelaide, South Australia, Australia; Cambridge University, United Kingdom

## Abstract

**Background:**

Uncertainty exists about benefits and harms of a planned vaginal birth after caesarean (VBAC) compared with elective repeat caesarean (ERC). We conducted a prospective restricted cohort study consisting of a patient preference cohort study, and a small nested randomised trial to compare benefits and risks of a planned ERC with planned VBAC.

**Methods and findings:**

2,345 women with one prior caesarean, eligible for VBAC at term, were recruited from 14 Australian maternity hospitals. Women were assigned by patient preference (*n* = 2,323) or randomisation (*n* = 22) to planned VBAC (1,225 patient preference, 12 randomised) or planned ERC (1,098 patient preference, ten randomised). The primary outcome was risk of fetal death or death of liveborn infant before discharge or serious infant outcome. Data were analysed for the 2,345 women (100%) and infants enrolled.

The risk of fetal death or liveborn infant death prior to discharge or serious infant outcome was significantly lower for infants born in the planned ERC group compared with infants in the planned VBAC group (0.9% versus 2.4%; relative risk [RR] 0.39; 95% CI 0.19–0.80; number needed to treat to benefit 66; 95% CI 40–200). Fewer women in the planned ERC group compared with women in the planned VBAC had a major haemorrhage (blood loss ≥1,500 ml and/or blood transfusion), (0.8% [9/1,108] versus 2.3% [29/1,237]; RR 0.37; 95% CI 0.17–0.80).

**Conclusions:**

Among women with one prior caesarean, planned ERC compared with planned VBAC was associated with a lower risk of fetal and infant death or serious infant outcome. The risk of major maternal haemorrhage was reduced with no increase in maternal or perinatal complications to time of hospital discharge. Women, clinicians, and policy makers can use this information to develop health advice and make decisions about care for women who have had a previous caesarean.

**Trial registration:**

Current Controlled Trials ISRCTN53974531

*Please see later in the article for the Editors' Summary*

## Abstract 2

A study conducted in Australia provides new data on the outcomes for mother and baby associated with either planned vaginal birth, or elective repeat caesarean section following a previous caesarean section.

## Editors' Summary

**Background:**

Rates of caesarean section are rising around the world, particularly in high- and middle-income countries, where most women have a choice of how their baby is delivered. Historically, the obstetrician in charge of the woman's care made the decision on whether to perform an elective (planned) caesarean section based on medical criteria. For women who have had a previous caesarean section, typically, their options for mode of childbirth are either a trial of vaginal birth or an elective repeat caesarean section. The proportion of women attempting a vaginal birth after a previous caesarean section has been declining in many countries partly due to the variable chance of achieving a successful vaginal birth (reported between 56% and 80%) and partly because of negative reports of the risk of complications, both to the mother and the baby, of a having a vaginal delivery following a caesarean section. Consequently, the rates of repeat caesarean section have risen sharply, for example, currently 83% in Australia and almost 90% in the US.

**Why Was This Study Done?:**

Both elective repeat caesarean section and subsequent vaginal delivery after a previous caesarean section have clinical risks and benefits. Most obviously, having a surgical procedure puts the woman having the repeat caesarean section at risk of surgical complications, especially if performed under a general anesthetic, and her baby may be at risk of respiratory complications. However, subsequent vaginal delivery following a previous caesarean section may put the mother at risk of bleeding severely enough to need a blood transfusion (more than 1,500 ml blood loss) and she may also be at increased risk of rupturing her uterus; and her baby may have an increased risk of dying or of becoming brain damaged due to lack of oxygen.

However, to date there have been no randomized controlled trials of elective repeat caesarean section versus vaginal delivery following a previous caesarean section to compare the health outcomes of mother and baby and a recent systematic review could draw no conclusions. So the researchers conducted this prospective cohort study based on patient preference (with a few women agreeing to be randomized to mode of delivery), to compare the health outcomes for mother and baby for elective repeat caesarean section versus vaginal delivery in women following a previous caesarean section.

**What Did the Researchers Do and Find?:**

Between 2002 and 2007, the researchers recruited 2,345 suitable women (that is, women who had one previous caesarean section, were currently 37 weeks pregnant with a single baby, and who were clinically able to have a vaginal delivery) from 14 maternity hospitals throughout Australia. A few women (22) agreed to be randomized to either mode of delivery but most women chose her preferred option. Then, depending on the woman's preferences for mode of birth, participating obstetricians either scheduled a date for an elective caesarean section (1,098 women) or assessed on-going suitability for the woman to have a planned vaginal delivery (1,225 women). However only 535 (43.2%) women who chose to have a vaginal birth were able to deliver this way because of failure to progress in labor or fetal distress: 334 of these women (27.0%) had to have an elective caesarean section and 368 women had to have an emergency caesarean section.

Although no women died, women who had a planned caesarean section experienced less severe bleeding than women who delivered vaginally. There were no infant deaths in those born by elective caesarean section but two unexplained stillbirths in the planned vaginal delivery group. There was also a reduced risk of nonfatal serious outcome before discharge from hospital for infants delivered by in the elective caesarean section. The researchers calculated that one infant death or near death would be prevented for every 66 elective caesarean sections performed in women who had a previous caesarean section.

**What Do These Findings Mean?:**

These findings show that in women who had delivered by a previous caesarean section delivering their next baby by planned caesarean section was associated with less infant death and better health outcomes for the mother before she was discharged from the hospital compared to women who had a subsequent vaginal delivery. This information can be used by women, clinicians, and policy makers in helping to make decisions about the mode of subsequent deliveries and best care for women who have had a previous caesarean section.

**Additional Information:**

Please access these Web sites via the online version of this summary at http://dx.doi.org/10.1371/journal.pmed.1001192.

- This study is linked to a *PLoS Medicine* Research Article by Fitzpatrick and colleagues and a *PLoS Medicine* Perspective by Catherine Spong
- The American Congress of Obstetricians and Gynecologists has information sheets for patients on caesarean sections and on vaginal birth after caesarean delivery
- Childbirth Connection, a US-based not-for-profit organization, provides information about caesarean sections and about vaginal birth after caesarean
- The National Childbirth Trust, a UK charity, provides information for parents on all aspects of pregnancy and birth, including caesarean sections and vaginal birth after caesarean delivery
- The UK charity Healthtalkonline has personal stories from women making decisions about birth after a caesarean section

## Introduction

Caesarean section is one of the commonest operations performed on childbearing women, with rates continuing to rise worldwide. For women who have had a previous caesarean, choices for mode of birth in their next pregnancy are either a trial of vaginal birth after caesarean (VBAC) or an elective repeat caesarean (ERC). For women who attempt a VBAC, the chance of achieving vaginal birth has been variably reported between 56% [1] and 80% [2]. The proportion of women attempting a VBAC has been declining in many countries [3], fuelled by negative reports of an increase in the risk of maternal and infant complications related to VBAC [4], including uterine rupture [5] and perinatal death [6]. The rates of repeat caesarean birth following a previous caesarean have risen commensurately, reaching 83% in Australia [7] and almost 90% in the US [3]. Repeat caesarean now accounts for 28% of all births in the United Kingdom [8].

Both ERC and VBAC have benefits and harms. Risks of planned VBAC when compared with planned ERC include haemorrhage, need for blood transfusion, endometritis, uterine rupture, perinatal death, and hypoxic ischaemic encephalopathy [8]–[12]. By comparison, women planning ERC are at increased risk of surgical complications, placenta accreta, and risks of multiple caesareans [11] and their infants are at risk of respiratory morbidity [10],[11]. Couples may experience subsequent infertility. [9]–[12].

There have been no randomised controlled trials comparing health outcomes after VBAC and ERC although the difficulty of conducting such trials has been recognised [9],[11]. A comprehensive systematic review of the nonrandomised literature comparing ERC with VBAC concluded that the current literature was “significantly flawed,” and that future research “should focus on comparability of the groups, specificity of the intervention, and standard outcome measures.” [10]. The need for evidence to inform women, clinicians, and policy makers about health outcomes of intended planned mode of birth rather than actual has been highlighted as critical [11]. To address these research gaps we conducted a prospective restricted cohort study that became effectively a patient preference study, with a smaller randomised trial (Text S1) [13] to compare the benefits and risks of a planned ERC with planned VBAC.

## Methods

### Ethics Statement

Ethics approval was granted by the Children's Youth and Women's Health Services Human Research Ethics Committee at the Women's and Children's Hospital, and by the local institutional review boards for each centre.

### Study Design and Participants

We conducted a multicentre study at 14 Australian maternity hospitals, all staffed and equipped to provide recommended care for VBAC or caesarean [12]–[17]. Women were eligible who had a single prior caesarean presenting in their next pregnancy with a live singleton in cephalic presentation, at 37 wk gestation or more, and who were considered eligible to attempt planned VBAC by their obstetrician (Text S1) [13]. Women were ineligible with more than one prior caesarean birth; a vertical, inverted T or unknown uterine incision; previous uterine rupture; previous uterine surgery (including hysterotomy or myomectomy involving entry of the uterine cavity or excessive myometrial dissection); previous uterine perforation; multiple pregnancy; any contraindication to vaginal birth (including placenta praevia, transverse lie, active genital herpes infection); cephalo-pelvic disproportion as judged by the clinician; lethal congenital anomaly; or fetal anomaly associated with mechanical difficulties at birth. The inclusion and exclusion criteria were based on clinical practice guidelines for eligibility for a VBAC [12],[14]–[17].

In the antenatal clinic eligible women were provided with written information about the randomised trial and patient preference study, pamphlets on VBAC and caesarean [18],[19], and asked if they would participate by the research officer. Recruitment started in November 2002 and was completed in May 2007.

### Randomisation, Masking, and Group Allocation

Women who gave written informed consent to the randomised trial, were randomly assigned to either planned VBAC or planned ERC, using a central telephone randomisation service. The randomisation schedule was prepared by an investigator not involved with clinical care, with stratification by centre and previous successful vaginal birth, using balanced variable blocks. Participants, staff, and investigators were not masked to treatment allocation. Women who gave written informed consent to the preference study were asked their preference for either planned VBAC or planned ERC, and were assigned to their preferred study group. Baseline information, including age, parity, marital status, body mass index (BMI), socioeconomic status (SES), psychological wellbeing, quality of life, and ethnicity self-reported by the participant was collected to compare the study groups.

### Interventions

**Planned VBAC group.** Participating clinicians agreed to follow the study protocol for intrapartum care for women in the planned VBAC group on the basis of clinical practice guidelines [12],[14]–[17],[20]–[22]. After study entry, women who planned to have a vaginal birth awaited spontaneous onset of labour. The attending obstetrician made assessment of the woman's on-going suitability for a planned VBAC.

**Planned ERC group:** Women who planned to have an ERC had this scheduled between 38 wk and 40 wk, preferably at 39 wk. If a woman in the planned ERC group entered labour prior to the scheduled elective surgical procedure, a caesarean was considered as an emergency.

### Outcomes

Study outcomes were important established measures of term infant morbidity and maternal morbidity, up to the time of primary hospital discharge after birth collected by trained research personnel. The primary prespecified outcome for this study was a composite of death or serious outcome for the infant defined as: death (any fetal death after study entry or death of a liveborn infant before hospital discharge [excluding lethal congenital anomalies]); or serious morbidity (defined as one or more of: birth trauma [subdural or intracerebral haemorrhage, spinal cord injury, basal skull fracture, other fracture, peripheral nerve injury present at discharge from hospital]); seizures at <24 h age or requiring two or more drugs to control; Apgar score <4 at 5 min; cord pH<7.0 (arterial or venous cord blood) and/or cord blood base deficit ≥12; neonatal encephalopathy stage 3; admission to the neonatal intensive care unit (NICU)>4 d; severe neonatal lung disease (defined as mean airway pressure >10 and or fraction of inspired oxygen >0.80 with need for ventilation); proven necrotising enterocolitis; and proven systemic infection in first 48 h of life treated with antibiotics.

Secondary study outcomes related to serious outcomes for the woman defined as one or more of: maternal death; uterine rupture (defined as a clinically significant rupture involving the full thickness of the uterine wall and requiring surgical repair); severe haemorrhage (blood loss of ≥1,500 ml and/or requiring blood transfusion); hysterectomy for any complications resulting from birth; vulvar or perineal haematoma requiring evacuation; deep vein thrombosis or thrombophlebitis requiring anticoagulant therapy; pulmonary embolus requiring anticoagulant therapy; pneumonia due to infection, aspiration or other causes; adult respiratory distress syndrome; wound infection (requiring prolongation of hospital stay or readmission) or wound dehiscence; damage to the bladder, ureter, or bowel requiring repair, or cervical laceration extending to the lower uterine segment, or abnormal extension of the uterine incision; occurrence of a fistula involving the genital tract; bowel obstruction or paralytic ileus; pulmonary oedema; stroke (defined as acute neurological deficit >24 h); cardiac arrest or respiratory arrest; any other serious maternal complication related to birth (as judged by the adverse events committee, while remaining masked to group allocation and mode of birth).

### Statistical Methods

Analyses were performed using intention-to-treat principles, according to the woman's assigned mode of birth at study entry, with the use of SAS software, version 9.1. Initial analyses were unadjusted. We prespecified that analyses would be adjusted for key prognostic variables with imbalance. We therefore adjusted analyses for maternal SES, BMI, and indication for previous caesarean birth where possible. Binary variables were analysed with log-binomial regression to give relative risks and 95% CIs. Continuous variables, if normally distributed, were analysed with a Student's *t*-test, and nonparametric tests were used for skewed data. We tested for interaction between treatment groups (VBAC and ERC) by study arm (randomised and patient preference) by calculating 95% CIs on the basis of the score method for the difference in treatment of the outcome proportions for binary data [23]. For all outcomes, apart from emergency caesarean where results are presented separately, no interactions were detected and the results of the preference and randomised arms were combined. The difference of proportions metric was used, as the number of women in the randomised arm was small generating zero cells. Consequently the relative risk was undefined for some outcomes. A *p*-value of less than 0.05 was considered to indicate statistical significance (2-sided). An interim analysis was not performed.

### Sample Size

We estimated that a sample size of 2,314 women would have statistical power of 80% (two-tailed alpha level 0.05) to detect an increase of 2% in the risk of fetal death or liveborn infant death prior to discharge or serious infant outcome from 1.6% for planned ERC [24] to 3.6% for planned VBAC (Text S1) [13].

## Results

**Figure 1:**
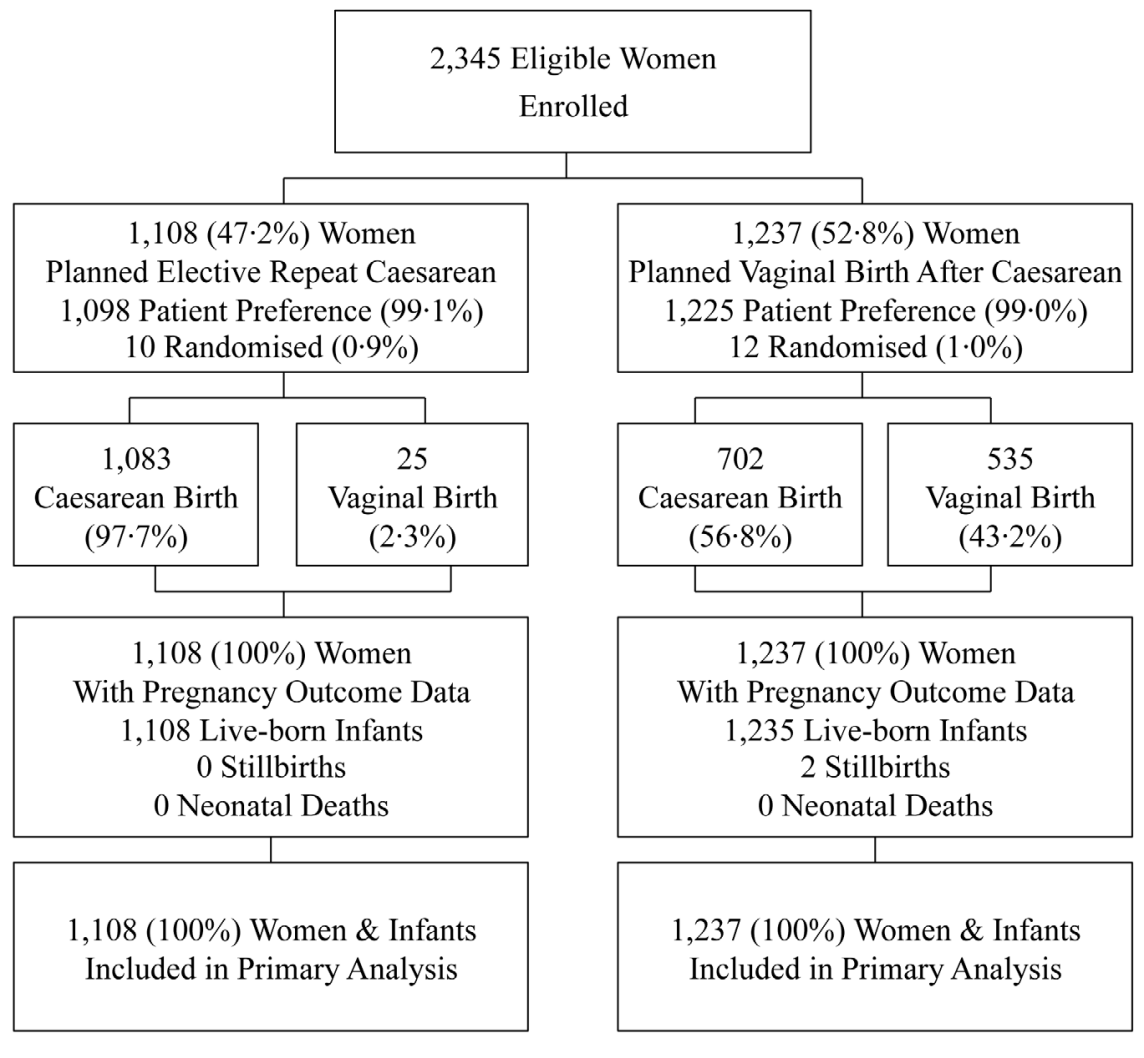
Flow of participants in the study. Data are numbers (%).

Of the 2,345 women enrolled, 1,108 (47.2%) were in the planned ERC group (ten randomised; 1,098 patient preference) and 1,237 (52.8%) in the planned VBAC group (12 randomised; 1,225 patient preference) (Figure 1). Clinical outcomes to primary hospital discharge after birth were available for all women and their infants.

The two treatment groups were similar at the time of study entry apart from women in the planned ERC group compared with women in the VBAC group being of slightly higher BMI, and SES, and there were some differences in the reasons for the previous caesarean section (Table 1).

**Table 1:** Baseline maternal characteristics.

Outcome	*n* (%), Mean ± SD, or Median and Interquartile Range					
	RCT		Patient Preference		Planned ERC Group (*n* = 1,108)	Planned VBAC Group (*n* = 1,237)
	Planned ERC (*n* = 10)	Planned VBAC (*n* = 12)	Planned ERC (*n* = 1,098)	Planned VBAC (*n* = 1,225)		
Age (y)	29.0±4.2	32.6±6.2	31.1±5.2	30.7±5.1	31.1±5.2	30.7±5.1
Gestational age entry (wk)	37.6 (37.0–38.0)	38.1 (37.3–39.1)	37.4 (37.1–38.2)	37.5 (37.1–38.4)	37.4 (37.1–38.2)	37.5 (37.1–38.4)
Previous vaginal birth	1 (10.0)	5 (41.7)	143 (13.0)	188 (15.3)	144 (13.0)	193 (15.6)
Smoker	1 (10.0)	3 (25.0)	177 (16.1)	194 (15.8)	178 (16.1)	197 (15.9)
Body mass index	23.1 (21.6–26.9)	28.5 (27.1–33.4)	26.7 (22.2–32.0)	25.9 (22.3–30.8)	27.9 (23.9–32.9)	26.8 (23.5–31.4)
Ethnicity^a^						
Caucasian	7 (70.0)	9 (75.0)	957 (87.2)	1,038 (84.7)	964 (87.0)	1,047 (84.6)
Asian	3 (30.0)	3 (25.0)	95 (8.7)	116 (9.5)	98 (8.8)	119 (9.6)
Other	0 (0.0)	0 (0.0)	46 (4.2)	71 (5.8)	46 (4.2)	71 (5.8)
Married/de facto	9 (90.0)	10 (83.3)	908 (82.7)	989 (80.7)	917 (82.8)	999 (80.8)
Socioeconomic index^b^						
Low SE index (disadvantaged)	1 (10.0)	1 (8.3)	256 (23.3)	341 (27.8)	257 (23.2)	342 (27.6)
Low-mid SE index	2 (20.0)	3 (25.0)	265 (24.1)	314 (25.6)	268 (24.2)	318 (25.7)
Mid-high SE index	7 (70.0)	2 (16.7)	210 (19.1)	240 (19.6)	218 (19.7)	241 (19.5)
High SE index	0 (0.0)	6 (50.0)	367 (33.4)	330 (26.9)	365 (32.9)	336 (27.2)
Emotional wellbeing						
EPDS Score	10.0 (3.0–13.0)	6.0 (5.0–14.0)	6.0 (3.0–9.0)	6.0 (3.0–10.0)	6.0 (3.0–9.0)	6.0 (3.0–10.0)
Depressed (EPDS>12)	3 (33.3)	4 (36.4)	111 (11.4)	121 (11.4)	114 (11.6)	125 (11.6)
Anxiety (STAI)	12.0±3.6	12.5±4.6	10.8±3.6	10.9±3.4	10.9±3.6	10.9±3.5
Physical functioning (SF36)	59.4±29	51.1±25	61.5±25	62.5±24	61.5±25.5	62.3±24.1
Role physical (SF36)	58.3±45	38.6±45	41.8±42	43.7±41	41.9±41.6	43.6±41.2
Bodily pain (SF36)	51.9±20	52.7±29	58.8±22	60.4±22	58.7±22.4	60.3±21.9
General health (SF36)	72.9±23	70.4±20	78.6±17	79.0±16	78.5±16.7	78.9±15.8
Vitality (SF36)	43.9±19	58.2±16	50.4±20	51.5±19	50.3±19.6	51.5±18.7
Social functioning (SF36)	73.6±18	78.4±26	76.7±22	76.8±22	76.7±21.9	76.8±21.9
Role emotional (SF36)	63.0±42	75.8±42	83.8±32	82.1±33	83.6±32.2	82.0±33.1
Mental health (SF36)	77.8±13	69.5±16	77.7±15	77.0±15	77.7±15.0	76.9±15.0
Physical component (SF36)	40.0±9.3	36.6±9.7	39.1±10	40.0±9.6	39.1±9.9	39.9±9.6
Mental Component (SF36)	48.7±9.5	51.8±9.6	52.5±9.0	52.1±9.0	52.5±9.0	52.1±9.0
Brazier health utility (SF36)	0.7±0.2	0.71±0.1	0.74±0.1	0.74±0.1	0.7±0.1	0.7±0.1
Main reasons for previous caesarean						
Failure to progress	1 (10.0)	5 (41.7)	469 (42.7)	394 (32.2)	470 (42.4)	399 (32.3)
Fetal distress	6 (60.0)	8 (66.7)	423 (38.5)	467 (38.1)	429 (38.7)	475 (38.4)
Breech	2 (20.0)	1 (8.3)	217 (19.8)	411 (33.6)	219 (19.8)	412 (33.3)
Cephalopelvic disproportion	1 (10.0)	1 (8.3)	81 (7.4)	64 (5.2)	82 (7.4)	65 (5.3)
Failed induction	2 (20.0)	1 (8.3)	46 (4.2)	59 (4.8)	48 (4.3)	60 (4.9)
Antepartum haemorrhage	1 (10.0)	1 (8.3)	42 (3.8)	60 (4.9)	43 (3.9)	61 (4.9)
Maternal choice	0 (0.0)	0 (0.0)	73 (6.6)	32 (2.6)	73 (6.6)	32 (2.6)
Maternal medical disorder	0 (0.0)	2 (16.7)	31 (2.8)	24 (2.0)	31 (2.8)	26 (2.1)
Pre-eclampsia/eclampsia	1 (10.0)	0 (0.0)	66 (6.0)	63 (5.1)	67 (6.0)	63 (5.1)
Other	1 (10.0)	1 (8.3)	65 (5.9)	63 (5.1)	66 (6.0)	64 (5.2)

a Ethnicity as reported by the participant.

b Socioeconomic index as measured by SEIFA where high index scores indicate increasing levels of social disadvantage.

EPDS, Edinburgh Postnatal Depression Scale [32]; SD, standard deviation; SE, socioeconomic; STAI, Spielberger State-Trait Anxiety Inventory [33]; SF3, SF36-Health Survey Questionnaire [34].

### Primary Outcomes

The risk of fetal or liveborn infant death prior to discharge or serious outcome was significantly reduced for infants born to women in the planned ERC group compared with infants of women in the planned VBAC group (planned ERC 10/1,108 [0.9%] versus planned VBAC 30/1,237 [2.4%], relative risk [RR] 0.39, 95% CI 0.19–0.80, *p* = 0.011) (Table 2). The number needed to treat to benefit with planned ERC to prevent fetal death or liveborn infant death prior to discharge or serious outcomes in one infant was 66 (95% CI 40–200).

**Table 2:** Primary outcomes.

Outcome	RCT				Patient Preference				Planned ERC Group (*n* = 1,108)		Planned VBAC Group (*n* = 1,237)		Adjusted RR (95% CI)^a^		*p*-Value
	Planned ERC (*n* = 10)		Planned VBAC (*n* = 12)		Planned ERC (*n* = 1,098)		Planned VBAC (*n* = 1,225)					
	*n*	Percent	*n*	Percent	*n*	Percent	*n*	Percent	*n*	Percent	*n*	Percent	CI	Range	
Death or serious infant outcome^b^	0	(0.0)	0	(0.0)	10	(0.9)	30	(2.4)	10	(0.9)	30	(2.4)	0.39	(0.19–0.80)	0.011
Perinatal death	0	(0.0)	0	(0.0)	0	(0.0)	2	(0.2)	0	(0.0)	2	(0.2)			0.50
Stillbirth	0	(0.0)	0	(0.0)	0	(0.0)	2	(0.2)	0	(0.0)	2	(0.2)			
Neonatal death	0	(0.0)	0	(0.0)	0	(0.0)	0	(0.0)	0	(0.0)	0	(0.0)			
Serious neonatal morbidity	0	(0.0)	0	(0.0)	10	(0.9)	28	(2.3)	10	(0.9)	28	(2.3)	0.41	(0.20–0.83)	0.014
Birth trauma	0	(0.0)	0	(0.0)	0	(0.0)	3	(0.2)	0	(0.0)	3	(0.2)			0.25
Seizures	0	(0.0)	0	(0.0)	1	(0.1)	1	(0.1)	1	(0.1)	1	(0.1)	1.11	(0.07–17.8)	1.00
Apgar ≤4 at 5 min	0	(0.0)	0	(0.0)	0	(0.0)	1	(0.1)	0	(0.0)	1	(0.1)			1.00
Cord pH<7.0	0	(0.0)	0	(0.0)	1	(0.1)	6	(0.5)	1	(0.1)	6	(0.5)	0.19	(0.02–1.54)	0.13
Cord blood base deficit ≥12 mmol/l	0	(0.0)	0	(0.0)	1	(0.1)	9	(0.7)	1	(0.1)	9	(0.7)	0.13	(0.02–1.06)	0.06
Stage 3 encephalopathy	0	(0.0)	0	(0.0)	0	(0.0)	0	(0.0)	0	(0.0)	0	(0.0)			
NICU admission >4 d	0	(0.0)	0	(0.0)	4	(0.4)	7	(0.6)	4	(0.4)	7	(0.6)	0.64	(0.18–2.25)	0.48
Severe lung disease	0	(0.0)	0	(0.0)	2	(0.2)	1	(0.1)	2	(0.2)	1	(0.1)	2.23	(0.20–24.6)	0.61
Necrotising enterocolitis	0	(0.0)	0	(0.0)	0	(0.0)	0	(0.0)	0	(0.0)	0	(0.0)			
Proven systemic infection	0	(0.0)	0	(0.0)	1	(0.1)	4	(0.3)	1	(0.1)	4	(0.3)	0.28	(0.03–2.49)	0.38

a Values adjusted for maternal SES, BMI, and indication for previous caesarean birth.

b Death or serious outcome includes: death (any fetal death after study entry or death of a liveborn infant prior to hospital discharge [excluding lethal congenital anomalies]); or serious neonatal morbidity (defined as one or more of: birth trauma [subdural or intracerebral haemorrhage, spinal cord injury, basal skull fracture, other fracture, peripheral nerve injury present at discharge from hospital]; seizures at <24 h age or requiring two or more drugs to control; Apgar score <4 at 5 min; cord pH<7·0; cord blood base deficit ≥12 mmol/l; neonatal encephalopathy stage 3; admission to NICU>4 d; severe neonatal lung disease [defined as MAP>10 and or FiO_2_>0·80 with need for ventilation]; proven necrotising enterocolitis; or proven systemic infection in first 48 h of life treated with antibiotics).

NICU, neonatal intensive care unit.

When the individual components of the primary endpoint were examined there was a statistically significant reduction in the risk of serious morbidity for infants born to women in the planned ERC group, compared with infants born to women in the planned VBAC group (planned ERC 10/1,108 [0.9%] versus planned VBAC 28/1,235 [2.3%], RR 0.41, 95% CI 0.20–0.83, *p* = 0.014). There were no statistically significant differences identified for any of the other individual components of the primary outcome between the two treatment groups.

No perinatal deaths occurred among infants of mothers in the planned ERC group. There were two stillbirths in the planned VBAC group. Both infants were born at 39 wk. The cause of death after autopsy for both infants was unexplained stillbirth.

### Secondary Outcomes

There were no maternal deaths in this study. The risk of maternal death or serious morbidity for women in the planned ERC group was 3.1% (34/1,108) and 4.5% (56/1,237) for women in the planned VBAC group; this was not a statistically significant difference (RR 0.69, 95% CI 0.46–1.05, *p* = 0.08). When the individual components of the composite outcome were examined, significantly fewer women in the planned ERC group had a major haemorrhage (defined as blood loss >1, 500 ml and/or need for blood transfusion) when compared with planned VBAC (planned ERC 9/1,108 [0.8%] versus planned VBAC 29/1,237 [2.3%], RR 0.37, 95% CI 0.17–0.80, *p* = 0.011, NNTB 66, 95% CI 40–187). There were no statistically significant differences between the treatment groups for any of the other secondary outcomes (Table 3).

**Table 3:** Secondary maternal outcomes by planned mode of birth.

Outcome	RCT				Patient Preference				Planned ERC Group (*n* = 1,108)		Planned VBAC Group (*n* = 1,237)		Adjusted RR (95% CI)^a^		*p*-Value
	Planned ERC (*n* = 10)		Planned VBAC (*n* = 12)		Planned ERC (*n* = 1,098)		Planned VBAC (*n* = 1,225)					
	*n*	Percent	*n*	Percent	*n*	Percent	*n*	Percent	*n*	Percent	*n*	Percent	CI	Range	
Serious maternal outcome^b^	0	(0.0)	0	(0.0)	34	(3.1)	56	(4.6)	34	(3.1)	56	(4.5)	0.69	(0.46–1.05)	0.08
Maternal death	0	(0.0)	0	(0.0)	0	(0.0)	0	(0.0)	0	(0.0)	0	(0.0)			
Uterine rupture	0	(0.0)	0	(0.0)	1	(0.1)	3	(0.2)	1	(0.1)	3	(0.2)	0.37	(0.04–3.57)	0.63
Major haemorrhage >1500 ml	0	(0.0)	0	(0.0)	9	(0.8)	29	(2.4)	9	(0.8)	29	(2.3)	0.37	(0.17–0.80)	0.011
Hysterectomy	0	(0.0)	0	(0.0)	0	(0.0)	1	(0.1)	0	(0.0)	1	(0.1)			1.0
Vulval/perineal haematoma	0	(0.0)	0	(0.0)	1	(0.1)	2	(0.2)	1	(0.1)	2	(0.2)	0.56	(0.05–6.15)	1.0
DVT requiring anticoagulation	0	(0.0)	0	(0.0)	1	(0.1)	0	(0.0)	1	(0.1)	0	(0.0)			0.47
PE requiring anticoagulation	0	(0.0)	0	(0.0)	0	(0.0)	0	(0.0)	0	(0.0)	0	(0.0)			
Pneumonia	0	(0.0)	0	(0.0)	0	(0.0)	0	(0.0)	0	(0.0)	0	(0.0)			
Adult respiratory distress	0	(0.0)	0	(0.0)	0	(0.0)	0	(0.0)	0	(0.0)	0	(0.0)			
Wound infection/dehiscence	0	(0.0)	0	(0.0)	18	(1.6)	13	(1.1)	18	(1.6)	13	(1.1)	1.62	(0.77–3.40)	0.20
Organ damage requiring repair	0	(0.0)	0	(0.0)	6	(0.5)	15	(1.2)	6	(0.5)	15	(1.2)	0.46	(0.17–1.20)	0.11
Genital tract fistula	0	(0.0)	0	(0.0)	0	(0.0)	1	(0.1)	0	(0.0)	1	(0.1)			1.0
Bowel obstruction/ileus	0	(0.0)	0	(0.0)	0	(0.0)	0	(0.0)	0	(0.0)	0	(0.0)			
Pulmonary oedema	0	(0.0)	0	(0.0)	0	(0.0)	1	(0.1)	0	(0.0)	1	(0.1)			1.0
Stroke	0	(0.0)	0	(0.0)	0	(0.0)	0	(0.0)	0	(0.0)	0	(0.0)			
Cardiac arrest	0	(0.0)	0	(0.0)	0	(0.0)	0	(0.0)	0	(0.0)	0	(0.0)			
Respiratory arrest	0	(0.0)	0	(0.0)	0	(0.0)	0	(0.0)	0	(0.0)	0	(0.0)			

a Values adjusted for maternal SES, BMI, and indication for previous caesarean birth.

b Death or serious maternal outcome includes: one or more of maternal death; uterine rupture (defined as a clinically significant rupture involving the full thickness of the uterine wall and requiring surgical repair); severe haemorrhage (blood loss of ≥1,500 ml and/or requiring blood transfusion); hysterectomy for any complications resulting from birth; vulval or perineal haematoma requiring evacuation; deep vein thrombosis (DVT) or thrombophlebitis requiring anticoagulant therapy; pulmonary embolus (PE) requiring anticoagulant therapy; pneumonia due to infection, aspiration or other causes; adult respiratory distress syndrome; wound infection (requiring prolongation of hospital stay or readmission) or wound dehiscence; damage to the bladder, ureter, or bowel requiring repair, or cervical laceration extending to the lower uterine segment, or abnormal extension of the uterine incision; occurrence of a fistula involving the genital tract; bowel obstruction or paralytic ileus; pulmonary oedema; stroke (defined as acute neurological deficit >24 h); cardiac arrest; respiratory arrest.

In the planned ERC group 1,083 (97.7%) women gave birth by caesarean section, with the majority (87.6%) as an elective procedure. In the planned VBAC group 535 (43.2%) women had a vaginal birth and 702 (56.8%) had a caesarean section; 334 (27.0%) as an elective and 368 (29.7%) as an emergency procedure (Table 4). The main indications for caesarean section in the planned VBAC group were previous caesarean section, failure to progress, and fetal distress. Women in the planned ERC group compared with women in the planned VBAC group gave birth at an earlier gestational age and although their median length of postnatal hospital stay was longer they were not more likely to stay more than 7 d (Table 4).

**Table 4:** Labour and birth outcomes.

Outcome	*n* (%), Mean ± SD, or Median and Interquartile Range						Adjusted RR (95% CI)^a^		*p*-Value
	RCT		Patient Preference		Planned ERC Group (*n* = 1,108)	Planned VBAC Group (*n* = 1,237)			
	Planned ERC (*n* = 10)	Planned VBAC (*n* = 12)	Planned ERC (*n* = 1,098)	Planned VBAC (*n* = 1,225)			CI	Range	
Gestational age at birth (wk)	39.2±0.7	40.0±1.0	38.8±0.7	40.0±1.1	38.8±0.7	40.0±1.1	−1.1	(−1.2 to 1.01)	<0.001
Induction of labour	0 (0.0)	2 (16.7)	4 (0.4)	151 (12.3)	4 (0.4)	153 (12.4)	0.03	(0.01–0.08)	<0.001
Mode of birth									
Vaginal birth	2 (20.0)	7 (58.3)	23 (2.1)	528 (43.1)	25 (2.3)	535 (43.2)	0.06	(0.04–0.09)	<0.001
Caesarean section	8 (80.0)	5 (41.7)	1,075 (97.9)	697 (56.9)	1,083 (97.7)	702 (56.8)	1.67	(1.59–1.75)	<0.001
Elective caesarean section	5 (50.0)	4 (33.3)	966 (88.0)	330 (26.9)	971 (87.6)	334 (27.0)	3.10	(2.82–3.42)	<0.001
Emergency caesarean section									
Preference arm			109 (9.9)	367 (30.0)	109 (9.9)	367 (30.0)	0.32	(0.27–0.40)	<0.001
Randomised arm	3 (30.0)	1 (8.3)			3 (30)	1 (8.3)	3.60	(0.44–29.5)	0.19
Analgesia/anaesthesia	9 (90.0)	12 (100)	1,080 (98.4)	1,148 (93.7)	1,089 (98.3)	1,160 (93.8)	1.04	(1.02–1.06)	<0.001
Epidural	3 (30.0)	4 (33.3)	183 (16.7)	425 (34.7)	186 (16.8)	429 (34.7)	0.47	(0.41–0.55)	<0.001
Postpartum haemorrhage >500 ml	2 (20.0)	2 (16.7)	184 (16.8)	214 (17.5)	186 (16.8)	216 (17.5)	0.91	(0.76–1.09)	0.30
Postpartum infection	0 (0.0)	0 (0.0)	4 (0.4)	3 (0.2)	4 (0.4)	3 (0.2)	1.49	(0.33–6.64)	0.71
Length of stay (d)	5 (3–5)	4 (3–5)	5 (4–6)	4 (3–5)	5 (4–6)	4 (3–5)			<0.001
Length of stay >7 d	0 (0.0)	0 (0.0)	60 (5.5)	62 (5.1)	60 (5.4)	62 (5.0)	1.08	(0.76–1.54)	0.67
Infant birthweight (g)	3,401±475	3,534±425	3,462±451	3,571±495	3,462±451	3,571±494	−56	(−252 to 139.4)	0.57
Apgar score <7 at 5 min	0 (0.0)	0 (0.0)	3 (0.3)	8 (0.7)	3 (0.3)	8 (0.6)	0.48	(0.12–1.86)	0.29

a Values adjusted for maternal SES, BMI, and indication for previous caesarean birth.

RCT, randomised controlled trial.

## Discussion

In this study involving women with a single prior caesarean who had reached 37 wk gestation in their next pregnancy, and who did not have a contraindication to a planned VBAC, a plan to birth by ERC was associated with a beneficial reduction in the risk of fetal death or liveborn infant death prior to discharge or serious outcome for the infant, when compared with women who planned VBAC. For women planning a VBAC our 2.4% risk of death or serious outcome for the infant is similar to that reported from previous cohort studies that compared actual rather than planned mode of birth where risks ranged between 0.13% and 2.4% [4],[9].

There are a number of strengths to our study design, enhancing the validity of our results. To our knowledge, this is the first randomised trial to report on health outcomes in this setting. However, few women consented to the randomised trial, as was suggested likely by our pre-trial survey of women's views [25]. Although the randomised controlled trial is regarded as the “gold standard” research methodology for assessing the effects of health care interventions, some research questions cannot be fully answered using this design, particularly where patients have strong treatment preferences, and decline randomisation as in our setting. Given our experience here and the recognised difficulty of recruitment to randomised trials related to VBAC [9],[11], it seems unlikely that large randomised trials will be conducted, although these may still be possible in other health care settings.

This is the first study designed around women's planned preferences for birth after caesarean, among women who were eligible for a VBAC, and therefore provides a high quality estimate of the benefits and harms associated with the two planned or intended treatment choices for birth, not previously reported. Most of the previous evidence has been based on comparison of actual mode of birth where groups were less comparable [11]. Information relating to health outcomes for women and their infants is known for all 2,345 women enrolled in the study, and our sample size was sufficiently large to allow us to detect important, small differences between the two policies for care.

Flaws identified in the literature related to the risks and benefits of planned ERC and planned VBAC have included a lack of comparability of groups, specifically being unclear whether women included in the ERC group were truly eligible to attempt VBAC [10]. Our study methodology ensured that all women recruited were considered eligible to attempt VBAC, on the basis of current clinical practice guidelines, as assessed by qualified staff at the time of study entry. We made a comprehensive assessment of known confounders with statistical adjustments for minor imbalances found between treatment groups for BMI, SES, and indication for previous caesarean. Nevertheless, unmeasured confounding may still account in part for the study findings. Our intention-to-treat analysis ensured that the study evaluated a policy of choice around planned ERC and planned VBAC.

We identified no increase in the risk of short-term maternal morbidity related to planned ERC, but rather a beneficial reduction in the risk of major maternal haemorrhage and/or the need for blood transfusion compared with planned VBAC. This finding is in sharp contrast to other reports where ERC has been associated with an increase in maternal blood loss [4],[26] and justifies further study.

The risk of symptomatic uterine scar rupture was low for both treatment groups being 0.1% for women in the planned ERC group and 0.2% for women in the planned VBAC group. This risk of uterine rupture related to VBAC is lower than that reported from the NICHD cohort study of 0.7% [4] and lower than the rate of symptomatic uterine scar rupture of 1.2/100 to 3.9/1,000 among women having a VBAC reported in systematic reviews of other cohort studies [9]–[11]. The standardised treatment schedules for VBAC and ERC, based on relevant evidence-based clinical practice guidelines used by all participating hospitals, may account for the low rates observed.

There is a well-documented increase in the risk of both perinatal mortality and infant morbidity with increasing gestational age beyond term [27],[28]. The differences in the risk of death or serious outcome for infants born to women in the planned VBAC group could be related to the difference in gestational age at birth observed between the two groups, rather than planned mode of birth. The relationship between advancing gestational age and morbidity and mortality, and the optimal time of birth for women at term therefore warrants further prospective evaluation.

Although statistically significant, overall the absolute risk difference in adverse health outcomes between the two forms of care remains small. Nevertheless, these small differences in the risk of short-term adverse health outcomes, either for the women or for their infants, are likely to be of considerable importance to the women, and therefore influence their choice of preferred mode of birth [29]. Absolute risk differences (related to mode of birth) may vary by factor, such as previous successful vaginal birth, where planned VBAC is more likely to result in vaginal birth. Similar proportions of women in each treatment group had achieved a previous vaginal birth prior to the caesarean (13% in the planned ERC group and 15.6% in the planned VBAC group). There have been no studies comparing the risks and benefits of VBAC with ERC that have reported on the health outcomes beyond the neonatal period. This lack of information on long-term health of either VBAC or ERC should be included in the counselling provided to women to assist with their decision making.

There is a need to establish whether the identified short-term benefits in health and wellbeing persist or are balanced by later risks. Therefore the evaluation of longer-term health, for both the women and children in this study, will be important. Our planned longer-term follow-up at early school age will assess maternal and child health as well as outcome in subsequent pregnancies including the risk from multiple caesareans, such as placenta praevia and accreta, and fertility [11],[12].

Our “restricted” prospective cohort study design used methodological features of high quality randomised trials, which included identification of a “zero time” for determining eligibility, study entry, and baseline characteristics; use of inclusion and exclusion criteria; treatment protocols derived from evidence-based clinical practice guidelines; standardised definitions for clinical outcomes; adjustment for imbalance in confounders at study entry; and the use of intention-to-treat analyses [30],[31].

Our results, whilst not generalisable for other populations, indicate that for women who have had one previous caesarean birth and are considered eligible at term to attempt a planned VBAC in their next pregnancy, an ERC as planned mode of birth is significantly associated with a lower risk of both fetal death or liveborn infant death prior to discharge or serious infant morbidity and major maternal haemorrhage without increasing other maternal and perinatal complications. Women, clinicians, and policy makers can use this information to develop health advice to assist in making evidence-based decisions about care for women who have had a previous caesarean and their infants.

## Supporting Information

Text S1 Protocol.
(PDF)

Text S2 CONSORT statement.
(PDF)

## Abbreviations

BMI: body mass index
ERC: elective repeat caesarean
SES: socioeconomic status
RR: relative risk
VBAC: vaginal birth after caesarean
