# Supplementary material for: Planned Vaginal Birth or Elective Repeat Caesarean: Patient Preference Restricted Cohort with Nested Randomised Trial
Source: PLoS Med. 2012 Mar 13;9(3):e1001192. doi: 10.1371/journal.pmed.1001192 (PMC3302845; doi:10.1371/journal.pmed.1001192)
Supplement: Text S1 — Protocol. (PDF) [file pmed.1001192.s001.pdf]

BMC Pregnancy and Childbirth

BioMed Central

Study protocol

Open Access

Birth after caesarean study – planned vaginal birth or planned elective repeat caesarean for women at term with a single previous caesarean birth: protocol for a patient preference study and randomised trial

Jodie M Dodd*[1], Caroline A Crowther[1], Janet E Hiller[2], Ross R Haslam[3] and Jeffrey S Robinson[1]

Address: [1]Discipline of Obstetrics and Gynaecology, The University of Adelaide, Adelaide, Australia, [2]Discipline of Public Health, The University of Adelaide, Adelaide, Australia and [3]Department of Perinatal Medicine, The Women's and Children's Hospital, Adelaide, Australia

Email: Jodie M Dodd* - jodie.dodd@adelaide.edu.au; Caroline A Crowther - caroline.crowther@adelaide.edu.au; Janet E Hiller - janet.hiller@adelaide.edu.au; Ross R Haslam - ross.haslam@cywhs.sa.gov.au; Jeffrey S Robinson - jeffrey.robinson@adelaide.edu.au

* Corresponding author

Published: 14 August 2007

BMC Pregnancy and Childbirth 2007, **7**:17 doi:10.1186/1471-2393-7-17

Received: 25 June 2007
Accepted: 14 August 2007

This article is available from: http://www.biomedcentral.com/1471-2393/7/17

© 2007 Dodd et al; licensee BioMed Central Ltd.
This is an Open Access article distributed under the terms of the Creative Commons Attribution License (http://creativecommons.org/licenses/by/2.0), which permits unrestricted use, distribution, and reproduction in any medium, provided the original work is properly cited.

Abstract

Background: For women who have a caesarean section in their preceding pregnancy, two care policies for birth are considered standard: planned vaginal birth and planned elective repeat caesarean. Currently available information about the benefits and harms of both forms of care are derived from retrospective and prospective cohort studies. There have been no randomised trials, and recognising the deficiencies in the literature, there have been calls for methodologically rigorous studies to assess maternal and infant health outcomes associated with both care policies.

The aims of our study are to assess in women with a previous caesarean birth, who are eligible in the subsequent pregnancy for a vaginal birth, whether a policy of planned vaginal birth after caesarean compared with a policy of planned repeat caesarean affects the risk of serious complications for the woman and her infant.

Methods/Design: Design: Multicentred patient preference study and a randomised clinical trial.

Inclusion Criteria: Women with a single prior caesarean presenting in their next pregnancy with a single, live fetus in cephalic presentation, who have reached 37 weeks gestation, and who do not have a contraindication to a planned VBAC.

Trial Entry & Randomisation: Eligible women will be given an information sheet during pregnancy, and will be recruited to the study from 37 weeks gestation after an obstetrician has confirmed eligibility for a planned vaginal birth. Written informed consent will be obtained. Women who consent to the patient preference study will be allocated their preference for either planned VBAC or planned, elective repeat caesarean. Women who consent to the randomised trial will be randomly allocated to either the planned vaginal birth after caesarean or planned elective repeat caesarean group.

Treatment Groups: Women in the planned vaginal birth group will await spontaneous onset of labour whilst appropriate. Women in the elective repeat caesarean group will have this scheduled for between 38 and 40 weeks.

Primary Study Outcome: Serious adverse infant outcome (death or serious morbidity).

Sample Size: 2314 women in the patient preference study to show a difference in adverse neonatal outcome from 1.6% to 3.6% (p = 0.05, 80% power).

Clinical Trial Registration: ISCTRN5397431

Background

Caesarean section is a common surgical procedure performed on women worldwide. The rate of caesarean section in most developed countries around the world has continued to increase over recent years, and currently accounts for 21.3% of all births in the United Kingdom, 23% in Northern Ireland [1], 23.3% in Australia [2], and 26% in the United States [3]. Caesarean section rates in South America are reported to be even higher, reaching in excess of 50% in some private hospitals in Chile, Argentina, Brazil and Paraguay [4]. Many reasons have been suggested to account for the increase in caesarean section observed over recent years, including the increasing use of electronic fetal heart rate monitoring during labour, a reduction in the training available to obstetricians in both operative vaginal births and vaginal breech births, in addition to fears of litigation [5].

Repeat caesarean section is the most common primary indication for a woman undergoing a repeat caesarean, accounting for 28% of births in the United Kingdom [1] and over 40% of births in the United States [6]. In South Australia, the main reason (56.6%) for women having an elective caesarean is that they have had a previous caesarean section, and 13.9% of emergency caesareans performed are in women who have had a previous caesarean [7]. Figures from the United States in 2003 indicate a repeat caesarean section rate of 89.4%, with a similar proportion of caesareans (88.7%) occurring in women considered to be 'low-risk' [8].

Many studies have examined the reasons for the increase in the proportion of caesarean births observed, assessing population characteristics, variations in clinician practice [9], available resources, women's childbirth preferences [9-12], and the views of health care practitioners [13-15]. The American College of Obstetricians and Gynecologists issued a consensus statement supporting vaginal birth after caesarean section as a "safe and acceptable" care option, in an attempt to address the increasing caesarean section rate, and increase the proportion of women attempting vaginal birth after caesarean [16].

While there is considerable variation in the proportion of women who are offered and attempt VBAC, successful rates of between 56% and 80% are reported in the literature [9,13,17,18]. British figures indicate that among women with a prior caesarean section, 33% will successfully achieve vaginal birth in a subsequent pregnancy, although again there was considerable variation across institutions, ranging from 6% to 64% [1]. Rates of planned VBAC across sub-Saharan Africa are reported between 54 and 97%, with successful vaginal birth being achieved in 63 to 84% of women [19].

Despite widespread attempts to increase the proportion of women with a previous caesarean who attempt VBAC [20], the number of women attempting VBAC has declined markedly. This is highlighted by data from the United States, indicating a fall in the number of women attempting VBAC from 28.3% in 1996 to 12.7% in 2002 [3]. Further contributing to this decline in the proportion of women attempting VBAC are recent literature reports highlighting an increase in both maternal and infant risks associated with VBAC, including uterine rupture [21-23] and perinatal death [24]. These reports may have accelerated the declining trend in VBAC rates [3].

Public interest and debate over the relative safety of vaginal birth after caesarean continues, with calls from key international agencies for better quality evidence [25].

Benefits and harms associated with vaginal birth after caesarean and elective repeat caesarean section

Both repeat elective caesarean section and VBAC are associated with benefits and harms. Repeat elective caesarean birth is associated with an increase in the risk of maternal complications such as bleeding, need for blood transfusion, infection, damage to the bladder and bowel, and deep venous thrombosis. As the number of caesarean births for each individual woman increases, so does the difficulty in performing surgery due to adhesions, and the risk of damage to the bladder or bowel at the time of surgery. There may also be difficulties in conceiving a further child or the development of placenta praevia or placenta accreta/percreta [26]. Infants born by caesarean may develop transient tachypnoea of the newborn, the risks of

Table 1: Clinical outcomes from the five identified systematic reviews

Outcome	Trial of Labour	Elective Repeat Caesarean
Febrile Morbidity		
- Rosen [32,33]	9.6/100	17.3/100
- Boulvain [19]	Not Separated	Not Separated
- Mozurkewich [31]	4.3/100	5.5/100
- Guise [25]	8.6–9.7/100	6.6–6.8/100
- Dodd [30]	Not Reported	Not Reported
Uterine Rupture/Dehiscence		
- Rosen [32,33]	1.8/100	1.9/100
- Boulvain [19]	Not Separated	Not Separated
- Mozurkewich [31]	3.9/1000	1.6/1000
- Guise [25]	2.7/1000	Not Reported
- Dodd [30]	1.2/100	0
Low Apgar Score		
- Rosen [32,33]	2.4/100	1.6/100
- Boulvain [19]	Not Separated	Not Separated
- Mozurkewich [31]	2.2/100	9/1000
- Guise [25]	Not Reported	Not Reported
- Dodd [30]	4.2/100	8/1000
Perinatal Death		
- Rosen [32,33]	1.8/100	1/100
- Boulvain [19]	Not Separated	Not Separated
- Mozurkewich [31]	5.8/1000	3.4/1000
- Guise [25]	1.3–9/1000	1/10 000–5/1000
- Dodd [30]	7.7/1000	0
Maternal Death		
- Rosen [32,33]	2.8/1000	2.4/10 000
- Boulvain [19]	Not Separated	Not Separated
- Mozurkewich [31]	0	0
- Guise [25]	Not Reported	Not Reported
- Dodd [30]	0	0

this relating to the use of general anaesthesia and gestational age at birth [27,28].

One uncommon, but potentially serious complication associated with a prior uterine surgery (including a previous caesarean section) is that of uterine rupture which may occur prior to the onset of labour, or during labour. Any vaginal birth may be associated with a non-reassuring fetal heart rate assessment, or failure to progress, both of which may require birth by emergency caesarean section. Emergency caesarean in labour is associated with an increased chance of infection, bleeding, and deep venous thrombosis, when compared with both vaginal birth and elective caesarean birth. Any vaginal birth may be associated with trauma to the woman's perineum and may be associated with longer-term problems, including pelvic floor weakness contributing to symptoms of prolapse and incontinence.

In searching the literature in preparation of a Cochrane Review [29] to evaluate the benefits and harms of vaginal birth after caesarean and elective repeat caesarean section for health outcomes for women and infants, in excess of 2000 case series and cohort studies, and five systematic reviews [19,25,30-35] were identified. The magnitude of risks for both repeat elective caesarean section and vaginal birth after caesarean section are best summarized through the published systematic reviews. While the methodology of each of the meta-analyses was well defined, the magnitude of the clinical outcomes reported varied considerably (see Table 1).

The most comprehensive of the systematic review was conducted by Guise and colleagues, in which 180 studies were identified, that compared the benefits and harms of a trial of labour and an elective repeat caesarean section [25,34,35]. In an assessment of study quality, the conclusion reached indicated the literature to be 'significantly flawed', with comparisons between studies hampered by poor standards of reporting, inconsistent definitions of outcomes, considerable variation in reporting of important clinical outcomes, and lack of comparability of groups, specifically it often being unclear whether women included in the elective repeat caesarean group were truly

eligible to attempt vaginal birth after caesarean [25,34,35].

The recommendations of the Guise systematic review highlighted the need for further research that 'should focus on conducting methodologically rigorous studies to provide direct evidence regarding the relative benefits and harms of VBAC and ERC. If randomised trials are not done, good-quality studies of VBAC versus ERC must pay attention to comparability of the groups, specificity of the intervention, and standard outcome measures'[25,34,35].

The National Institute of Child Health and Human Development Maternal Fetal Medicine Units Network recently conducted a large prospective observational study across 19 centres in the United States [36]. A total of 33,699 women with a prior caesarean birth were involved in the study, of whom 17,898 (53.1%) attempted VBAC, and 15,801 (46.9%) underwent elective repeat caesarean section. The observed rate of symptomatic uterine scar rupture among women attempting a VBAC was 0.7%. The absolute risk of infant hypoxic ischaemic encephalopathy was 0.46 per 1,000 women attempting a VBAC at term. The authors concluded that an attempted VBAC was associated with an increase risk of perinatal morbidity and mortality when compared with elective repeat caesarean section, although the absolute risks remained small [36].

Women's preferences for mode of birth

Women's expectations for birth and mode of birth preferences are influenced not only by knowledge of the potential benefits and risks but also personal and social factors. In the caesarean section audit conducted in the United Kingdom, 45% of women with a previous caesarean indicated a preference for vaginal birth in a subsequent pregnancy, while 20% preferred an elective repeat caesarean. While a further 27% of women had their preference determined by medical factors, only 6.2% of women expressed no preference for mode of birth [1]. In a survey of recent mothers who had given birth by caesarean section, 63% of women indicated a preference for their subsequent mode of birth (either VBAC or repeat elective caesarean section), with these preferences for care being formed within six months of the index caesarean birth [37].

In a systematic review, Eden and colleagues [38] identified that women with a previous vaginal birth were more likely to select a trial of labour in a subsequent pregnancy when compared with women who had not had a previous vaginal birth. The cited reasons included ease of recovery and need to return quickly to ongoing family responsibilities, rather than concerns primarily for the woman's own safety or that of her infant [38]. Any future research designed to influence the rate of planned vaginal birth after caesarean section must include an assessment of women's views, and an evaluation of women's process of decision making [25].

Justification for a randomised trial and patient preference study of planned VBAC versus planned repeat caesarean

To date, there have been no randomised controlled trials comparing VBAC with elective repeat caesarean section [29], and the observational studies currently available have limitations. Although the randomised controlled trial is regarded as the 'gold standard' research methodology for assessing the effects of health care interventions [39], some research questions cannot be fully answered using this design, particularly in situations where patients may have strong treatment preferences and thus decline randomisation [40,41].

Patient preferences have been effectively incorporated into a prospective cohort study design previously [42]. The inclusion of women with clear preferences for treatment improves generalisability of the study results [43], and this design has been used successfully in other studies relating to women's health issues [44,45].

Previous cohort studies relating to options for birth after a previous caesarean have been criticised for their striking methodological deficiencies, with strong recommendation that future studies avoid the following pitfalls: lack of comparability of groups, specifically the ERC group not guaranteed to be eligible for VBAC; failure to consider other important baseline comparability factors, poor specificity of the intervention and the importance of co-interventions, and lack of precise and standard outcome measures [25]. In addition the recommendation was that future studies should assess women's views of care and evaluate women's process of decision-making [25]. To specifically address these recommendations, the current research utilises rigorous methodology, using a 'restricted' prospective cohort study and a randomised clinical trial.

A 'restricted' prospective cohort study design utilises the rigour of recruitment, treatment schedules and follow up of the randomised controlled trial [46]. Adoption of this methodology has not been shown to systematically overestimate the magnitude of the treatment effects when compared with the results of randomised controlled trials [47]. Features of importance in the restrictive cohort design [46] include the identification of a 'zero time' for determining a woman's eligibility and baseline features; use of inclusion and exclusion criteria similar to those of clinical trials; adjustment for differences in base-line susceptibility to the outcomes; and the use of statistical methods (eg intention-to-treat analysis) similar to randomised controlled trials.

Using this methodology, we will conduct a, patient preference study and a randomised clinical trial to compare the benefits and risks of a planned VBAC with planned elective, repeat caesarean. Eligible women who consent to be randomised will be entered into the randomised trial. Eligible women who decline randomisation and consent to be entered into the patient preference study will choose their treatment preference and follow the study protocol for that treatment group.

Hypotheses

The primary null hypothesis is that for women who meet the accepted eligibility criteria for planned VBAC, there is no difference in the risk of death or serious adverse outcome up to the time of primary hospital discharge for the infant in women who have a planned VBAC compared with a planned elective repeat caesarean section.

The secondary null hypotheses are that for women, who meet the accepted, eligibility criteria for a planned VBAC, for women who have a planned VBAC compared with planned elective repeat caesarean there is no difference in the risk of serious maternal outcomes up to the time of primary hospital discharge.

Methods/Design

Study Design

Multicentred patient preference study and a randomised clinical trial.

Inclusion Criteria

Women with a single prior caesarean presenting in their next pregnancy with a single, live fetus in cephalic presentation, who have reached 37 weeks gestation, and who do not have a contraindication to a planned VBAC are eligible.

Exclusion Criteria

Women with any of the following are ineligible: more than one prior caesarean birth, vertical, inverted T or unknown uterine incision, previous uterine rupture, previous uterine surgery (including hysterotomy or previous myomectomy involving entry of the uterine cavity or excessive myometrial dissection), previous uterine perforation, multiple pregnancy, any contraindication to vaginal birth (including placenta praevia, transverse lie, active genital herpes infection), cephalo-pelvic disproportion as judged by the clinician, lethal congenital anomaly, fetal anomaly associated with mechanical difficulties at birth (such as hydrops, fetal ascites, hydrocephalus, omphalocele or cystic hygroma).

The inclusion/exclusion criteria are based on guidelines recommended by the Society of Obstetricians and Gynecologists of Canada [48], American College of Obstetrics and Gynecology [16], the Institute for Clinical Systems Improvement [49], and the National Institute for Clinical Effectiveness [50], UK.

Trial Entry

Ethical approval for this study has been obtained from each of the participating collaborating hospitals. Women at the collaborating hospitals who may be eligible for the study will be given the study information sheet during their pregnancy and a copy of the Royal Australian and New Zealand College of Obstetricians and Gynaecologists information pamphlets 'Vaginal birth after caesarean section – a guide for women' and 'Caesarean birth' [51,52]. Women will discuss their birth options with their primary caregiver.

Written information that contains a brief summary of the benefits and risks of vaginal birth and elective repeat caesarean given to women with a prior caesarean has been shown to be as effective as a more detailed prenatal education and support program on the proportion of women having a planned VBAC [53]. To maximise the likelihood that women will receive their planned choice for birth care or care allocated at randomisation, eligible women can be recruited from 37 weeks gestation onwards. As it is important to ensure that women in the 'restricted' prospective study who choose a planned elective repeat caesarean would have been eligible for a planned VBAC [25] an obstetrician will confirm that the woman is eligible for a planned vaginal birth at the time of study entry.

After the woman consents to be in the patient preference study or the randomised trial, and written, informed consent given to a member of the research team, entry details will be recorded on the entry form. During a short telephone call to the central telephone randomisation service, information will be given to check eligibility, to assist in follow-up, and to assist in the analysis of results. An investigator not involved with clinical care will prepare the randomisation schedule for the randomised study. Stratification at randomisation will be by collaborating centre, and previous successful vaginal birth.

Women consenting to the randomised trial will be randomised to either planned vaginal birth after caesarean or planned elective repeat caesarean, from the computer randomisation schedule and given a study number. Once all entry details are given at telephone recruitment and eligibility is confirmed, women consenting to the patient preference study will be asked their preference for either planned VBAC or planned, elective repeat caesarean, and given a study number.

At study entry, socio-demographic characteristics of the woman, details of previous caesarean section, and infor-

mation to assist in follow-up and analysis will be collected. Women will be asked to complete questionnaires to assess emotional wellbeing as measured by anxiety (Spielberger State-Trait Inventory Self Evaluation Questionnaire [54], and the six-item short form [55]), depression (Edinburgh Postnatal Depression Scale, [56]), quality of life (SF36-Health Survey Questionnaire [57]), the women's preference for either treatment policy and information about their decision-making process [58].

Treatment Groups

Planned Vaginal Birth After Caesarean Group

Women who plan to have a vaginal birth or are randomised to this care group will await the spontaneous onset of labour, whilst appropriate. At the time of onset of labour the woman should be reassessed as to her eligibility for a planned VBAC. If a complication arises necessitating caesarean section (eg fetal distress) or if exclusion criteria for a vaginal birth occur after study entry (eg malpresentation or suspected cephalopelvic disproportion due to increased fetal size) a caesarean should be undertaken. The guidelines for intrapartum care for women in the planned VBAC group are based on those recommended by the SOGC [48], ACOG [16], RCOG.) [14,59] and NICE [50]. Other aspects of care will follow the institutional guidelines at the hospital. Hospitals agreed to follow the following care recommendations.

Induction of labour

The need for induction of labour for medical or obstetric indications will be assessed and determined by the obstetrician caring for the woman. If induction is considered necessary, this will be performed according to the local hospital guidelines where the woman has planned to give birth. The judicious use of oxytocin is not contraindicated in the presence of a previous caesarean but requires close monitoring [16]. The guidelines issued by the RCOG on induction of labour in women with a prior caesarean highlight a sparsity of data specifically reporting the use of prostaglandin preparations in women with a previous caesarean, thereby precluding an ability to make specific recommendations for clinical care.) [59]. The risk of uterine rupture is higher after an induced labour and highest after induction with prostaglandins [22]. Prostaglandin E_2 gel for induction is therefore not recommended for use for women in this study.

Augmentation of labour

The judicious use of oxytocin for augmentation of labour is not contraindicated in the presence of a prior caesarean section but requires close monitoring [16,48].

Fetal heart rate monitoring

All women undergoing a planned VBAC are advised to have continuous fetal heart rate monitoring as recommended in the RCOG guidelines for the use of electronic fetal heart rate monitoring [14]. The presence of a non-reassuring fetal heart rate tracing will be managed by performing fetal scalp pH sampling when clinically possible, or emergency caesarean as appropriate [14].

Analgesia/Anaesthesia

Analgesia and anaesthesia should be available according to the woman's choice. Epidural analgesia may be used as requested [16].

Planned Elective Repeat Caesarean Group

Women who plan to have an elective repeat caesarean or are randomised to this care group will have this scheduled for between 38 weeks and 40 weeks (preferably at 39 weeks), with care provided according to the policy of the institution involved. The caesarean should be undertaken with appropriate skill and expertise for surgical procedures and analgesia/anaesthesia. If a woman in the planned elective repeat caesarean group goes into labour prior to the scheduled elective surgical procedure, a caesarean will be considered as an emergency according to the institutional guidelines.

Care during the antenatal period, labour and the postnatal stay of both groups

In order to meet the above requirements for care of women participating in the study, each hospital should be able to perform continuous fetal heart rate monitoring in labour, be able to perform fetal scalp pH for an abnormal fetal heart rate trace, have on-site skilled obstetric, anaesthetic and paediatric staff, able to perform emergency caesarean section, have available a consultant obstetrician for emergency back-up, and be able to cross match blood [48,49]. Care for the woman and baby will be by the primary caregivers.

Follow up after birth until the time of primary hospital discharge for both groups

After birth information will be obtained relating to birth and infant outcomes from the woman and infant's case notes. The birth form will be completed after the woman has given birth. Similarly the postnatal and neonatal forms will be completed for live born infants after discharge of both mother and baby from hospital.

Longer-term follow up for both groups:

Longer-term follow up of all women is currently planned to assess maternal physical and emotional health, wellbeing and child development.

Primary Study Endpoints

Serious adverse outcomes for the infant defined as:

Perinatal/Neonatal Mortality (defined as any fetal death after study entry or death of a liveborn infant within 28 days of age (excluding lethal congenital anomalies); or Serious neonatal morbidity (defined as one or more of the following, excluding lethal congenital anomalies: birth trauma (subdural or intracerebral haemorrhage, spinal cord injury, basal skull fracture, other fracture, peripheral nerve injury present at discharge from hospital); seizures at <24 hours age or requiring two or more drugs to control; Apgar score <4 at 5 minutes; cord pH<7.18; base deficit <-8 (arterial or venous cord blood); neonatal encephalopathy stage 3 (Sarnat & Sarnat 1976); admission to the neonatal intensive care unit (NICU) > 4 days; severe neonatal lung disease (defined as MAP >10 and or FiO_2 >0.80 with need for ventilation); proven necrotising enterocolitis; proven systemic infection in first 48 hours of life treated with antibiotics).

Definitions of adverse outcome are those used by the Australian and New Zealand Neonatal Network [60] and those considered by experts as important measures of term and post-term neonatal morbidity [61].

Secondary Study Endpoints

1. Serious adverse outcome for the woman is defined as one or more of the following: maternal death; uterine rupture (defined as a clinically significant rupture involving the full thickness of the uterine wall and requiring surgical repair); haemorrhage (blood loss of greater than 1500 mL and/or requiring blood transfusion); hysterectomy for any complications resulting from birth; vulvar or perineal haematoma requiring evacuation; deep vein thrombosis or thrombophlebitis requiring anticoagulant therapy; pulmonary embolus requiring anticoagulant therapy; pneumonia due to infection, aspiration or other causes; adult respiratory distress syndrome; wound infection (requiring prolongation of hospital stay or readmission) or wound dehiscence; damage to the bladder, ureter or bowel requiring repair, or cervical laceration extending to the lower uterine segment, or abnormal extension of the uterine incision; occurrence of a fistula involving the genital tract; bowel obstruction or paralytic ileus; pulmonary oedema; stroke (defined as acute neurological deficit >24 hours); cardiac arrest; respiratory arrest; any other serious maternal complication related to birth (as judged by the adverse events committee, while remaining blinded to group allocation and mode of birth).

Definitions of serious maternal outcomes are based on those considered as important outcome measures of maternal morbidity from The Term Breech Trial [62] and from the ASSHP consensus statement [63].

Sample Size

The incidence of serious adverse infant outcome is the primary endpoint of the study. The true risk of serious adverse infant outcome is estimated as 1.6% for planned elective, repeat caesarean birth [62]. It is proposed that sufficient women are recruited to provide reliable evidence about the effects of a policy of planned VBAC compared with a policy of planned elective, repeat caesarean on serious adverse infant outcomes. The sample size will detect a change in risk from 1.6% to 3.6% as suggested by the systematic reviews [31]. If the ratio of ERC to VBAC is 1:1 a study of 2180 women would have an 80% power of detecting a statistically significant difference at an alpha level of 0.05 (two tailed). Our pilot study has suggested a ratio of 5.5:4.5. Allowing for an ERC to VBAC ratio of 6.5:3.5 and a small loss to follow up (1%) the sample size required will be 2314 women.

The expected rate of serious maternal complications for planned elective repeat caesarean section is likely to be higher than the 3.9% found in the planned caesarean section arm of the Term Breech Trial [62]. Systematic reviews of the cohort studies of VBAC compared with elective repeat caesarean section provide an estimate of the risks for serious maternal complications of 7.7% [31]. A sample size of 2314 women will have 80% power (2-tailed alpha error of 0.05) of finding the following increases in risk for serious maternal complications: 16% to 20.6%, 12% to 16.1%, 8% to 11.5%, 6% to 9.1% and 4% to 6.7%.

Analysis and Reporting of Results

The initial analysis will examine the baseline characteristics of all women in the study, as an indication of comparability of treatment groups. These characteristics will include maternal age, race, height, weight, smoking history, vaginal birth prior to caesarean section, indication for previous caesarean section and treatment preference. Any differences in these prognostic variables that have treatment imbalance of sufficient magnitude will be controlled for in subsequent analyses. Log binominal regression will be used to adjust binary outcomes for confounders and linear regression for normally distributed outcomes. Comparison of outcomes for women and infants will be analysed for the primary and secondary outcomes on an 'intention to treat' basis, according to their planned care at recruitment. The relative risks and 95% confidence intervals will be reported for the major study outcomes, and the number needed to treat to benefit and the number needed to treat to harm will be calculated.

Competing interests

The author(s) declare that they have no competing interests.

Authors' contributions

JMD, CAC, JEH, RRH, and JSR all contributed to the development of the study protocol. JMD prepared the initial draft of this manuscript and all authors reviewed critically for content and approved the final version to be submitted.

Acknowledgements

This trial has been supported through the Australian National Health and Medical Research Council (NHMRC) in a three-year project grant (ID349460), and the Women's and Children's Hospital Foundation.

JMD is the recipient of the NHMRC Neil Hamilton Fairley Clinical Fellowship (ID399224).

References

1. RCOG: **The Sentinel National Caesarean Section Audit Report- RCOG Clinical Effectiveness Support Unit.** RCOG Press; 2001.
2. AIHW: **Australia's mothers and babies 2000.** In *Perinatal Statistics Series Number 12* Sydney, ; 2003.
3. Hamilton BE, Martin JA, Sutton PD: **Birth: Preliminary data for 2002.** *Natl Vital Stat Rep* 2003, **51**:4-5.
4. Belizan J, Althabe F, Barros F, Alexander S: **Rates and implications of cesarean sections in Latin America: ecological study.** *British Medical Journal* 1999, **319**:1397-1402.
5. Mastrobattista JM: **Vaginal birth after cesarean delivery.** *Obstetrics and Gynecology Clinics of North America* 1999, **26**:295-304.
6. Curtin SC, Kozak LJ, Gregory KD: **U.S. cesarean and VBAC rates stalled in the mid-1990's.** *Birth* 2000, **27**:54-57.
7. Chan A, Scott J, Nguyen A, Keane R: **Pregnancy outcome in South Australia, 2000.** Adelaide, Pregnancy Outcome Unit, Epidemiology Branch, Department of Human Services; 2001.
8. Menacker F: **Trends in cesarean rates for first births and repeat cesarean rates for low-risk women: United States, 1990-2003.** *Natl Vital Stat Rep* 2005, **54**:1-8.
9. Appleton B, Targett C, Rasmussen M, Readman E, Sale F, Permezel M: **Vaginal birth after caesarean section: an Australian multicentre study.** *Aust NZ J Obstet Gynaecol* 2000, **40**:87-91.
10. Gamble JA, Creedy DK: **Women's preferences for a caesarean section.** *Birth* 2001, **28**:101-110.
11. Quinlivan JA, Petersen RW, Nichols CN: **Patient preference the leading indication for elective caesarean section in a teaching hospital.** *Aust NZ J Obstet Gynaecol* 1999, **39**:207-214.
12. Turnbull DA, Wilkinson C, Yaser A, Carty V, Svigos JM, Robinson JS: **Women's role and satisfaction in the decision to have a caesarean section.** *Med J Aust* 1999, **170**:580-583.
13. Stone C, Halliday J, Lumley J, Brennecke S: **Vaginal birth after caesarean (VBAC): a population study.** *Paediatr Perinat Epidemiol* 2000, **14**:340-348.
14. Royal College of Obstetricians & Gynaecologists (RCOG): **Evidence-based clinical guideline number 8: the use of electronic fetal monitoring.** RCOG Press; 2001.
15. Appleton B, Targett C, Rasmussen M, Readman E, Sale F, Permezel M: **Knowledge and attitudes about vaginal birth after caesarean section in Australian hospitals.** *Aust NZ J Obstet Gynaecol* 2000, **40**:195-199.
16. American College of Obstetricians and Gynecologists (ACOG): **ACOG Practice Bulletin: vaginal birth after previous cesarean delivery. Clinical management guidelines.** *Int J Gynecol Obstet* 1999, **66**:197-204.
17. McMahon MJ: **Vaginal birth after cesarean.** *Clinical Obstetrics and Gynecology* 1998, **41**:369-381.
18. Flamm BL: **Once a cesarean, always a controversy.** *Obstet Gynecol* 1997, **90**:312-315.
19. Boulvain M, Fraser WD, Brisson-Carroll G, Faron G, Wollast E: **Trial of labour after caesarean section in sub-Saharan Africa: a meta-analysis.** *BJOG* 1997, **104**:1385-1390.
20. McIlwaine G., Boulton-Jones C, Cole S, Wilkinson C: **Caesarean section in Scotland 1994: a national audit.** *Scottish programme for Clinical Effectiveness in Reproductive Health* 1995.
21. Hibbard JU, Ismail MA, Wang Y, Te C, Karrison T, Ismail MA: **Failed vaginal birth after a cesarean section: how risky is it?** *Am J Obstet Gynecol* 2001, **184**:1365-1373.
22. Lydon-Rochelle M, Holt VL, Easterling TR, Martin DP: **Risk of uterine rupture during labor among women with a prior cesarean delivery.** *NEJM* 2001, **345**:3-8.
23. Sachs BP, Kobelin C, Castro MA: **The risks of lowering the caesarean delivery rate.** *NEJM* 1999, **340**:54-57.
24. Smith GCS, Pell JP, Cameron AD, Dobbie R: **Risk of perinatal death associated with labor after previous cesarean delivery in uncomplicated term pregnancies.** *JAMA* 2002, **287**:.
25. Guise JM, McDonagh M, Hashima J, al : **Vaginal birth after cesarean (VBAC). Evidence Report/Technology Assessment: Number 71.** *Agency for Healthcare Research and Quality* 2003.
26. Hemminki E, Merilainen J: **Long-term effects of cesarean sections: ectopic pregnancies and placental problems.** *Am J Obstet Gynecol* 1996, **174**:1569-1574.
27. Morrison JJ, Rennie JM, Milton PJ: **Neonatal respiratory morbidity and mode of delivery at term: influence of timing of elective caesarean section.** *British Journal of Obstetrics and Gynaecology* 1995, **102**:101-106.
28. Hook B, Kiwi R, Amini SB, Fanaroff A, Hack M: **Neonatal morbidity after elective repeat cesarean section and trial of labor.** *Pediatrics* 1997, **100**:348-353.
29. Dodd JM, Crowther CA, Huertas E, Guise JM, Horey D: **Planned elective repeat caesarean versus planned vaginal birth for women with a previous caesarean birth (Cochrane Review).** In *The Cochrane Library, Issue 4* Chichester, UK, John Wiley & Sons Ltd; 2004.
30. Dodd JM, Crowther CA: **Vaginal birth after caesarean versus elective repeat caesarean for women with a single prior caesarean section: a systematic review of the literature.** *Aust NZ J Obstet Gynaecol* 2004, **(in press)**:.
31. Mozurkewich EL, Hutton EK: **Elective repeat cesarean delivery versus trial of labor: a meta-analysis of the literature from 1989 to 1999.** *Am J Obstet Gynecol* 2000, **183**:1187-1197.
32. Rosen MG, Dickinson JC: **Vaginal birth after cesarean: a meta-analysis of indicators for success.** *Obstet Gynecol* 1990, **76**:865-869.
33. Rosen MG, Dickinson JC, Westhoff CL: **Vaginal birth after cesarean: a meta-analysis of morbidity and mortality.** *Obstet Gynecol* 1991, **77**:465-470.
34. Guise JM, Berlin M, McDonagh MS, Osterwell P, Chan BK, Helfand M: **Safety of vaginal birth after cesarean: a systematic review.** *Obstet Gynecol* 2004, **103**:420-429.
35. Guise JM, McDonagh MS, Osterwell P, Nygren P, Chan BK, Helfand M: **Systematic review of the incidence and consequences of uterine rupture in women with previous caesarean section.** *British Medical Journal* 2004, **329**:403.
36. Landon MB, Hauth JC, Leveno KJ, Spong CY, Leindecker S, Varner MW, Moawad AH, et al for the National Institute of Child Health and Human Development Maternal Fetal Medicine Units Network.: **Maternal and perinatal outcomes associated with trial of labor after prior cesarean delivery.** *NEJM* 2004, **351**:2581-2589.
37. Dodd JM, Pearce E, Crowther CA: **Women's experiences and preferences following caesarean birth.** *Aust NZ J Obstet Gynaecol* in press.
38. Eden KB, Hashima JN, Osterwell P, Nygren P, Guise JM: **Childbirth preferences after cesarean birth: a review of the evidence.** *Birth* 2004, **31**:49.
39. National Health and Medical Research Council (NHMRC): **Guidelines for the development and implementation of clinical practice guidelines.** 1999.
40. McPherson K, Britton A: **The impact of patient treatment preferences on the interpretation of randomised controlled trial.** *Eur J Cancer* 1999, **35**:1598-1602.
41. Torgerson D, Sibbald B: **Undertaking controlled trials: What is a patient preference trial?** *BMJ* 1998, **316**:360.
42. Brewin C, Bradley C: **Patient preferences and randomised clinical trials.** *BMJ* 1989, **299**:313-315.
43. Solomon MJ, McLeod RS: **Surgery and the randomised controlled trial: past, present and future.** *BMJ* 1998, **169**:380-383.
44. Cooper KG, Grant AM, Garratt AM: **The impact of using a partially randomised, patient preference design when evaluating alternative management for heavy menstrual bleeding.** *BJOG* 1997, **104**:1367-1373.

45. Henshaw RC, Majii SA, Russell IT, Templeton AA: **Comparison of medical abortion and surgical vacuum aspiration: women's preferences and acceptability of treatment.** *BMJ* 1993, **307:**714-717.
46. Horwitz R, Viscoli C, Clemens J, Sadock R: **Developing improved observational methods for evaluating therapeutic effectiveness.** *American Journal of Medicine* 1990, **89:**630-680.
47. Concato J, Shah N, Horwitz RI: **Randomized controlled trials, observational studies and the hierarchy of research designs.** *NEJM* 2000, **342:**1887-1892.
48. Society of Obstetricians and Gynaecologists of Canada: **SOGC Clinical Practice Guidelines: Guidelines for vaginal birth after previous caesarean birth.** *Int J Gynecol Obstet* 2005, **89:**319-331.
49. Institute for Clinical Systems Improvement: **Vaginal birth after cesarean.** 2001.
50. National Institute of Clinical Effectiveness (NICE): **Caesarean section.** London, RCOG Press; 2004.
51. Royal Australian and New Zealand College of Obstetricians and Gynaecologists (RANZCOG): **Vaginal birth after caesarean section - a guide for women.** 2002.
52. Royal Australian and New Zealand College of Obstetricians and Gynaecologists (RANZCOG): **Caesarean section - a guide for women.** 2002.
53. Fraser W, Maunsell E, Hodnett E, Moutquin JM: **Randomized controlled trial of a prenatal vaginal birth after cesarean section education and support program. Childbirth Alternatives Post-Cesarean Study Group.** *Am J Obstet Gynecol* 1997, **176:**419-425.
54. Spielberger CD: **The state-trait anxiety inventory.** Consulting Psychologists Press, Inc.; 1970.
55. Marteau TM, Bekker H: **The development of a six item form of the State Scale of the Spielberger State-Trait anxiety Inventory STAI.** *Brit J Clin Psychol* 1992, **31:**301-306.
56. Cox JL, Holden JM, Sagousky R: **Detection of postnatal depression development of the 10 item Edinburgh Depression Scale.** *BMJ* 1987, **150:**782-786.
57. Ware JE, Sherbourne CD: **The MOS 36-item short-form health survey (SF-36). Conceptual Framework and Item Selection.** *Med Care* 1992, **30:**473-483.
58. O'Connor AM: **Validation of a decisional conflict scale.** *Med Decis Making* 1995, **15:**25-30.
59. NICE (National Institute of Clinical Excellence): **RCOG Induction of labour. Evidence Based Clinical Guideline Number 9.** *National Institute of Clinical Excellence* 2001.
60. Donoghue D, Bawden K, Cartwright D, Darlow B, Henderson-Smart D, Lancaster P: **The report of the Australian and New Zealand Neonatal Network, 2000.** Sydney, ANZNN; 2002.
61. Hannah ME, Hannah WJ, Hellmann J: **Induction of labor as compared with serial antenatal monitoring in post-term pregnancy. A randomised controlled trial.** *NEJM* 1992, **326:**1587-1592.
62. Hannah ME, Hannah WJ, Hewson S.A., al : **Planned cesarean section versus planned vaginal birth for breech presentation at term: a randomised multicentre trial.** *Lancet* 2001, **356:**1375-1383.
63. Brown MA, Hague WM, Higgins J: **The detection, investigation and management of hypertension in pregnancy.** *Aust NZ J Obstet Gynaecol* 2000, **4:**139-155.

Pre-publication history

The pre-publication history for this paper can be accessed here:

http://www.biomedcentral.com/1471-2393/7/17/prepub

Publish with **BioMed Central** and every scientist can read your work free of charge

"BioMed Central will be the most significant development for disseminating the results of biomedical research in our lifetime."

Sir Paul Nurse, Cancer Research UK

Your research papers will be:

- available free of charge to the entire biomedical community
- peer reviewed and published immediately upon acceptance
- cited in PubMed and archived on PubMed Central
- yours — you keep the copyright

Submit your manuscript here:
http://www.biomedcentral.com/info/publishing_adv.asp

BioMedcentral
